# Sensorimotor Difficulties Are Associated with the Severity of Autism Spectrum Conditions

**DOI:** 10.3389/fnint.2016.00028

**Published:** 2016-08-17

**Authors:** Penelope Hannant, Sarah Cassidy, Teresa Tavassoli, Francesca Mann

**Affiliations:** ^1^Centre for Research in Psychology, Behaviour and Achievement, Coventry UniversityCoventry, UK; ^2^Seaver Autism Centre, Icahn School of Medicine, Mount SinaiNew York, NY, USA; ^3^School of Education, University of BirminghamBirmingham, UK

**Keywords:** autism, sensory, motor, sensorimotor, receptive language, social communication

## Abstract

Present diagnostic criteria for autism spectrum conditions (ASC) include social communication and interaction difficulties, repetitive behavior and movement, and atypical sensory responsivity. Few studies have explored the influence of motor coordination and sensory responsivity on severity of ASC symptoms. In the current study, we explore whether sensory responsivity and motor coordination differences can account for the severity of autistic behaviors in children with ASC. Thirty-six children participated: 18 (13 male, 5 female) with ASC (ages 7–16: mean age = 9.93 years) and 18 (7 male, 11 female) typically developing (TD) children (ages 6–12; mean age = 9.16 years). Both groups completed a battery of assessments that included motor coordination, sensory responsivity, receptive language, non-verbal reasoning and social communication measures. Children with ASC also completed the Autism Diagnostic Observation Schedule (ADOS) and Autism Diagnostic Interview—Revised (ADI-R). Results showed that children with ASC scored significantly lower on receptive language, coordination, sensory responsivity and a sensorimotor subscale, Modulation of Activity (MoA) compared to the TD group. In the ASC group, MoA significantly predicted ASC severity across all ASC measures; receptive language and sensory responsivity significantly predicted parental reported autism measures; and coordination significantly predicted examiner observed reported scores. Additionally, specific associations were found between the somatosensory perceptive modalities and ASC severity. The results show that sensorimotor skills are associated with severity of ASC symptoms; furthering the need to research sensorimotor integration in ASC and also implying that diagnosis of ASC should also include the assessment of both coordination deficit and atypical sensory responsivity.

## Introduction

Social communication is pivotal to both a child’s development and integration into society. Yet, those with an Autism Spectrum Condition (ASC) do not find it easy to communicate, interact and socialize with others: often finding it difficult to infer, intonate and interpret others’ behaviors. For this reason, current diagnostic criteria for ASC (DSM-5, American Psychiatric Association, 2013) incorporate difficulties in social communication and interaction, as well as sensory responsivity and motor movements. Current DSM-5 criteria (American Psychiatric Association, 2013) refers to Autism as a “disorder”, however in the current study, we use the less stigmatizing term “condition”, acknowledging that Autism includes strengths as well as weaknesses, while still being a medical condition for which individuals need support. Much research has attempted to uncover the reasons behind the social and communicative difficulties in ASC, and has identified correlations between severity of ASC and either motor coordination or sensory responsivity separately (Ming et al., 2007; Tomchek and Dunn, 2007; Green et al., 2009; Caminha and Lampreia, 2012). More recently, research has begun to look in greater detail at the role sensory responsivity and motor coordination difficulties play together in ASC through sensorimotor integration (Dowd et al., 2012; Siaperas et al., 2012; Gowen and Hamilton, 2013). Sensorimotor integration can be defined as “a brain process that allows, by complex neural operations, the execution of a certain voluntary motor behavior in response to specific demands of the environment” (Machado et al., 2010). This study therefore simultaneously investigates whether sensory and motor differences predict the social communication and interaction difficulties seen in autism.

Unusual movements and sensory difficulties are associated with ASC. In addition to stimming and rocking, general “clumsiness” has been reported in individuals with ASC since the first pioneering reports describing this condition (Kanner, 1943; Asperger, 1944; DeMyer, 1976; Damasio and Maurer, 1978). This has been further documented in more recent research. For example, motor skill scores for children with ASC often fall 1.5 standard deviations below the typical mean (Staples and Reid, 2010; MacNeil and Mostofsky, 2012), and approximately 80% have definite motor impairment with 10% being borderline (Miyahara et al., 1997; Green et al., 2009; Fournier et al., 2010; Kopp et al., 2010; Whyatt and Craig, 2012; Gowen and Hamilton, 2013). Coordination difficulties in ASC are evident from early infancy (Page and Boucher, 1998; Ming et al., 2007; Kopp et al., 2010), with parents being significantly more likely to report motor delays as the first concern at an average age of 14.7 months (Teitelbaum et al., 1998). Initial clinical reports also described sensory “disturbances” in ASC (Kanner, 1943; Asperger, 1944). Studies indicate the occurrence of atypical sensory responsivity in ASC is high, with 69–95% showing sensory responsivity dysfunction (Matson et al., 2010; Bhat et al., 2011). Research has also demonstrated the presence of a variety of sensory responsivity impairments in ASC (Chawarska et al., 2007; Caminha and Lampreia, 2012), such as; hyper and hypo responsivity across tactile (Baranek and Berkson, 1994; Blakemore et al., 2006); visual (Nyström et al., 2015); olfactory (Suzuki et al., 2003) and auditory domains (Madsen et al., 2014; Takahashi et al., 2014; Tavassoli et al., 2014b); differences in proprioceptive impairment (the sense of relative position in space) (Tavassoli et al., 2014b); and perceptual function (Paton et al., 2012).

Difficulties in coordination and/or sensory responsivity appear to be highly prevalent in people with ASC. However, are difficulties in these domains associated with severity of ASC symptoms, particularly social and communication skills? Research suggests that motor coordination difficulties impact on a number of skills, which are key for effective social participation. For example, individuals with ASC have significant impairments in skilled motor gestures such as imitation (Mostofsky et al., 2006) and the development of speech sound production (Page and Boucher, 1998). Children who have motor difficulties, without a diagnosis of ASC, tend to show weaker empathy for others (Cummins et al., 2005) and show increased anxiety on the playground (Bhat et al., 2011). There is also evidence that early motor difficulties are key to the development of ASC; children who show fine motor difficulties in early childhood (from 7 months old) have a significantly increased risk of developing an ASC by 36 months (Landa and Garrett-Mayer, 2006). This research suggests that motor difficulties are not only highly prevalent in ASC, but might impact social and communication skills in those with and without ASC.

Research has also identified links between sensory responsivity and the severity of ASC symptoms. For example, a number of studies have shown that sensory responsivity impairments impact social interaction and communication skills (Hilton et al., 2007; Reynolds et al., 2011; Matsushima and Kato, 2013). A more specific example is shown in research by Fitzgibbon et al. (2013), who proposed that as physical and social pain appear to be processed differently in individuals with ASC, their insensitivity to pain could consequently limit the understanding and empathy for pain in others. Studies have also shown correlations between self-reported sensory over-responsivity and autistic traits in adults with ASC (Tavassoli et al., 2014b), and sensory disturbance and autism severity (Kern et al., 2007; Ben-Sasson et al., 2009; Boyd et al., 2010; Lane et al., 2010; Tavassoli et al., 2014a). Hence, difficulties in sensory responsivity have also been associated with severity of ASC symptoms, particularly social and communication skills.

Difficulties in motor coordination and sensory responsivity are highly prevalent in ASC, are each associated with severity of ASC symptoms, and there is preliminary evidence that early motor coordination difficulties increase risk of developing ASC (Landa and Garrett-Mayer, 2006). However, these studies have explored each of these domains separately, despite them being innately connected (Brooks, 1983). For example, sensory feedback is fundamental to planning and executing the movement of reaching for a cup (such as being aware of your own position both visually and proprioceptively; Brooks, 1983; Gowen and Hamilton, 2013). Any miscalculations (such as missing the cup) during the movement are then processed and modified accordingly. Movements repeated in this way then create a feedforward program that predicts the sensory consequences of the action. Movements become procedural and automatic, thereby reducing the delay of continuous sensory feedback (Wolpert and Flanagan, 2001; Todorov and Jordan, 2002). For this reason deficiencies in sensory guidance are likely to affect both the ability to acquire and modify a stored motor command, leading to limited flexibility and accuracy. This strongly suggests that sensory responsivity and motor coordination should not be seen as distinct separate entities, but as a functional unit in the form of sensorimotor integration.

The high levels of comorbidity of ASC with sensory and motor difficulties that appear to impact adaptive functioning, in addition to their presence from birth (Brisson et al., 2012; Nyström et al., 2015), suggest that sensorimotor integration could play a key role in the development and maintenance of ASC rather than being just a symptom. Work conducted by Gowen and Hamilton (2013) support this theory; suggesting that atypical sensory input and variability in motor deployment “together” may play a crucial role in ASC. Additionally, a number of studies have shown a relationship between sensory responsivity and motor coordination delays in ASC. For example, using a cluster analysis to examine data from the Short Sensory Profile (McIntosh et al., 1999) and Vineland Adaptive Behavior Scales (Sparrow et al., 1984), Lane et al. (2010) suggested that motor coordination delays in ASC could be linked to under-responsivity in the vestibular sensory system. Glazebrook et al. (2009) demonstrated that adults with ASC had difficulty coordinating both hand and eye movements and Milne et al. (2006) found a significant relationship between visual motion responsivity and fine motor control by measuring both motion and form coherence in ASC. Gowen and Miall (2005) also identified impairments in the ability to integrate motor control successfully with sensory input by testing visually guided movement, speed complex movement, muscle tone, prediction, coordination and timing in ASC. Iwanaga et al. (2000) highlighted weaker sensorimotor integration in ASC by assessing sensorimotor functioning in higher functioning autism and autism participants using the Japanese version of the Miller Assessment for Preschoolers (Miller and Schouten, 1988). Difficulties integrating visual cues and other sensory feedback from the environment with motor movements have also been demonstrated (e.g., see Gowen et al., 2008; Dowd et al., 2012; Whyatt and Craig, 2012; Stins et al., 2015). Furthermore, specific areas of the brain associated with sensorimotor integration such as the cerebellum (Paulin, 1993; Glickstein, 1998) and the basal ganglia (Nagy et al., 2006; Chukoskie et al., 2013) have also shown abnormalities in ASC. For example 95% of autistic cerebella examined at autopsy showed clearly defined anatomic irregularities; most commonly a significantly decreased number of Purkinje cells, a large inhibitory neuron thought to regulate motor function (Bauman and Kemper, 2005; Amaral et al., 2008; Fatemi et al., 2012). A decreased volume in the basal ganglia has also been reported in ASC (Estes et al., 2011) in addition to the striatum, one of the largest components of the basal ganglia, having excess functional connectivity in ASC (Di Martino et al., 2011).

These studies have demonstrated that motor coordination and sensory responsivity are both key elements in ASC: often occurring together, working together and potentially influencing behavior together. Such difficulties could affect the necessary sensorimotor skills involved in non-verbal language and communication such as gesture and imitation, consequently leading to what is perceived as idiosyncratic behaviors i.e., eye gaze aversion, limited non-verbal communication and restricted facial gesture. These atypical behaviors in infancy could subsequently impact social learning opportunities, with a cascade effect on development of effective social communication skills (see Hannant et al., 2016, for a review).

Although research has already identified associations between either motor coordination or sensory responsivity with ASC severity in isolation, this study aims to replicate and extend these findings by looking at both these elements together. This will help future studies identify a more definitive point of difficulty within the sensorimotor chain in ASC. Specifically whether motor coordination, sensory responsivity and specifically sensorimotor integration (i.e., the neurology and connections between the two single domains) are associated with the severity of ASC symptoms. This study therefore explores whether: (1) there is a significant difference in sensory responsivity and motor coordination between children with and without ASC; and if (2) the level of sensory responsivity and motor coordination difficulties are concurrently associated with and can predict autism severity.

## Materials and Methods

### Participants

The ASC group was comprised of 18 children, (13 male, 5 female) aged 7–16 (mean age = 9.9 years) and were recruited from local ASC support groups in Warwickshire, UK. Sixteen children with ASC had a pre-existing diagnosis of ASC from a trained clinician according to DSM-IV criteria. The remaining two children with ASC were currently being referred for a clinical diagnosis of ASC. ASC diagnosis was confirmed by the research team using the Autism Diagnostic Observation Schedule General—2nd Edition (ADOS-2; Rutter et al., 2012) and the Autism Diagnostic Interview—Revised (ADI-R; Rutter et al., 2005), administered by a research reliable rater.

The typically developing (TD) group was comprised of 18 children (7 male, 11 female), aged 6–12 (mean age = 9.2 years), recruited during a research event held at Coventry University, UK, involving a number of different research studies. The TD group was recruited to this event by advertising in the local media in Warwickshire, UK. The TD group included children with no disabilities or diagnoses.

Participants completed: a parent report measure of autistic traits (Social Communication Questionnaire, SCQ; Rutter et al., 2003), to ensure that no children in the TD group had significantly high levels of autistic traits; a parent report measure of sensory responsivity (Sensory Profile, SP; Dunn, 1999); a measure of visual motor integration (VMI; Beery and Beery, 2010); and a measure of motor coordination (Movement Assessment Battery for Children, MABC; Henderson et al., 2007). Standard measures of intelligence such as the full scale Wechsler Abbreviated Scale of Intelligence (WASI; Wechsler and Hsiao-pin, 2011), which require a spoken response, appear to underestimate the abilities of children with autism (Bodner et al., 2014). Therefore, in the current study we matched participants in each group by age and using only the non-verbal intelligence quotient (IQ) matrices subset of the WASI (Wechsler and Hsiao-pin, 2011) and measured receptive language IQ using the British Picture Vocabulary Scale III (BPVS-III; Dunn et al., 2009). Groups differed significantly on receptive language ability, and there was a marginally insignificant between group difference in non-verbal IQ (despite the non-verbal IQ range being similar between groups; Table 1). Therefore both receptive language and non-verbal IQ are included as covariates in the correlation and supplementary regression analysis reported below. There was a significant group difference in gender ratio (Table 1), however, there was no effect of gender on sensory responsivity or motor coordination measures in either the ASC group (MABC *t*_(16)_ = 0.29, *p* = 0.78; SP *t*_(16)_ = 0.31, *p* = 0.76) or the TD group (Movement ABC *t*_(16)_ = 0.18, *p* = 0.86; SP *t*_(16)_ = 0.10, *p* = 0.92). The TD group scored significantly lower on parent reported autistic traits than the ASC group (*t*_(24)_ = 7.33, *p* = 0.000, *d* = 0.831), and no participants in the TD group scored above cut off indicating ASC on the SCQ (15). See Table 1 for characteristics of both groups.

**Table 1:** **Demographic descriptives and group comparisons**.

Group	Gender	Age in years	Non-verbal reasoning
ASC	13 M	9.93 ± 2.71	90.94 ± 13.28
(*N* = 18)	5 F		(71–112)
Control	7 M	9.16 ± 1.89	99.50 ± 12.68
(*N* = 18)	11 F		(70–117)
Difference	*X*^2^(1,18) = 4.05,	*t*_(34)_ = −1.00,	*t*_(34)_ = −1.98,
	*p* = 0.044*	*p* = 0.325	*p* = 0.056

Note: *denotes p < 0.05 Bonferroni Correction p = 0.025.

### Materials

Participants completed a battery of assessments, four of which were standardized (MABC, BEERY, BPVS, WASI) where a standardized score of 84 or below was considered below average and 115 or above considered above average. The other four assessments (ADOS-II, ADI-R, SCQ, SP) were criterion based with a given cut-off point. In this study raw scores were used on the MABC, in order to analyze findings in greater detail from each subset. The ADOS-II and ADI-R ASC measures were utilized in order to ensure full and robust measurement of ASC symptomology within the study. The SCQ was used as a measure of ASC symptomology across both the TD and ASC groups.

*The Movement Assessment Battery for Children—2* (MABC 2; Henderson et al., 2007): This is a standardized assessment of motor coordination for children aged 3–16 years and is a revision of the Test of Motor Impairment (TOMI; Stott et al., 1984). It is comprised of three components: manual dexterity, ball skills, static and dynamic balance. Examples of test content include placing pegs onto a board, throwing a beanbag onto a target and walking heel to toe along a line. The MABC 2 was normed on 1172 children aged 3–16 years with and without disabilities. Internal Reliability includes internal consistency estimates (range = 0.92–1.00) and validity with the “Draw-a-Man” test = 0.66 (Kavazi, 2006).

*The Beery-Buktenica Developmental Test of Visual-Motor Integration, Sixth Edition* (BEERY VMI; Beery and Beery, 2010): This is a standardized measure of an individual’s ability to combine visual perception (VP) and fine motor coordination for people aged 2–100 years. It is comprised of three parts: VMI, VP and fine motor coordination. The VMI assessment requires an individual to copy a series of developmentally progressive geometric shapes; the VP aspect involves identifying matching shapes; and the motor coordination subtest contains a variety of shape outlines that the individual draws lines within. The BEERY VMI (6th Edn.) was normed on 1737 individuals aged 2–18 years with and without disabilities, showing good inter-rater reliability (range = 0.993−0.98) and validity (range = 0.8−0.95; Beery and Beery, 2010).

*Sensory Profile* (SP; Dunn, 1999): A standardized parent report questionnaire for children aged 3–10 years, that assesses the frequency of a child’s responses to differing sensory modulation, processing and emotional events itemized in 125 questions. The SP consists of three domains: a sensory responsivity domain, which includes auditory, visual, vestibular, tactile, oral and multi-sensory processing; a modulation domain, which includes modulating sensory responsivity with relation to endurance and tone, proprioception (body position), movement affecting activity, emotional responses and visual filtering; and a behavioral emotional response domain which considers the behavioral outcomes of sensory responsivity. Higher scores on the SP indicate behaviors closer to the norm, or average. Lower scores indicate greater deviation from the norm, and thus greater difficulties in a particular area of sensory responsivity. The SP was normed on 1187 children aged 3–14 years of age with and without disabilities. Internal reliability includes internal consistency estimates (range = 0.47−0.91) and convergent and discriminant validity was determined by demonstrating high correlations with scores on the school function assessment (Dunn, 1999). “Modulation of Movement Affecting Activity Level” (MoA) is a sub division of the SP that specifically measures the child’s demonstration of activeness, and is considered a measure of the modulation of movement in relation to sensory responsivity or sensorimotor integration.

*British Picture Vocabulary Scale—Third Edition* (BPVS-III; Dunn et al., 2009): A standardized non-reading assessment of receptive language. Each item within the assessment consists of identifying the correct image out of four pictures provided, to match a given word that covers a range of subjects, such as verbs, animals, emotions, toys and attributes. The BPVS-III was normed on 1480 children aged 3–16 years with and without disabilities. Internal reliability = 0.91 and validity with the Wechsler (2005) Intelligence Scale for Children = 0.76 (Dunn et al., 2009).

*The Social Communication Questionnaire—Lifetime* (SCQ; Rutter et al., 2003): A standardized parent-report measure of autistic traits for children from 4 years of age. The lifetime form is composed of 40 yes or no questions and is used as a screening tool indicating whether referral for diagnosis of ASC is warranted. Scores of 15 or above out of 40 indicate a possible diagnosis of ASC. Scores therefore provided an index of the number of ASC symptoms an individual exhibits, with a higher score indicating higher levels of autistic traits. Items include questions based on reciprocal social interaction, such as friendships, reciprocal conversation and patterns of behavior. The SCQ was normed on 214 children aged 2–18 years with and without disabilities. Internal reliability = 0.84−0.93 and validity with the ADI-R = 0.78 (Rutter et al., 2003).

*Wechsler Abbreviated Scale of Intelligence—2nd Edition* (WASI-II; Wechsler and Hsiao-pin, 2011); A brief standardized measure of verbal and non-verbal intelligence. The matrices subset was used in the current study to measure non-verbal reasoning in both groups. The WASI was normed on approximately 2900 individuals aged 6–90 years with and without disabilities. Matrix Internal Reliability = 0.87−0.94 and validity with the WRIT = 0.71 (Wechsler and Hsiao-pin, 2011).

The following diagnostic measures were also completed by the ASC group to independently confirm participants ASC diagnosis and indicate severity of ASC symptoms.

*Autism Diagnostic Observation Schedule—2nd Edition* (ADOS-II; Rutter et al., 2012): A standardized diagnostic instrument for diagnosis of ASC, and confirmation of ASC diagnosis for research purposes. It consists of a semi-structured interview that provides a number of social presses and opportunities to code quality of social and communicative behaviors. The 2nd edition of the ADOS also includes a rating indicating the severity of ASC symptoms taking into account the person’s age and expressive language level. The ADOS-II was validated on 381 individuals aged between 15 months to 40 years with and without disabilities, with a further 1139 children aged between 14 months to 16 years recruited to revise the algorithms. Inter-rater reliability showed over 80% agreement on all modules with a high level of discriminative validity between autism and TD resulting in specificities of 50–84 and sensitivities of 91–98 (Rutter et al., 2012).

*Autism Diagnostic Interview—Revised* (ADI-R; Rutter et al., 2005): A standardized diagnostic instrument for diagnosis of ASC, and confirmation of ASC diagnosis for research purposes. It consists of a detailed semi-structured interview to gather evidence from an informant (parent, sibling or partner of an individual) on an individual’s current behavior and early development indicative of an ASC diagnosis. Interviews cover social and communication, repetitive stereotyped behaviors, sensory and motor skills, talents, and challenging behaviors. The ADI-R was validated on 50 children aged between 36–59 months with and without disabilities. Internal Reliability demonstrated 26 of 35 weighted kappa values were 0.70 or higher. The ADI-R also shows a high level of discriminative validity with Clinical Diagnosis with 24 out 25 children being correctly diagnosed using the ADI-R (Rutter et al., 2005).

### Procedure

Ethical approval for the study was obtained from the local research ethics committee. After parental consent to take part in the study was obtained, the parent completed the ADI-R either over the phone or in person with a researcher who was research reliable in both ADI-R and ADOS-II. The parent and child were then invited to a single assessment session at the University. During this session, the following assessments were carried out in random order, to counterbalance and combat order affects, by trained researchers: BEERY VMI, Movement ABC, ADOS-II, BPVS and WASI non-verbal subsets. During this time the participant’s parent also completed the SP and SCQ. Before the assessment procedure each task was explained carefully and depending on autism severity, a visual timetable produced to help alleviate anxiety. During the test procedure each participant was invited to have a voluntary break after each assessment.

## Results

### Analysis Approach

Data were analyzed using SPSS (version 22), and normality tests conducted using Skewness and Kurtosis outputs. All measured variables: ADI-R, ADOS, SCQ, MABC Total, BEERY VMI, Sensory Profile Total, MoA, BPVS III and Matrices did not deviate significantly from normal (*z*-scores were all <1.96). Following tests for normality, TD and ASC data (BPVS-III, MABC, SP, MoA, SCQ, BEERY VMI) were compared using Bonferroni corrected independent *t*-tests. *Post hoc* tests were then completed in order to identify specific components of the MABC and SP that differed between groups significantly. Cohen’s *d* is used as an indicator of effect size, with 0.2 indicating a small, 0.5 medium and 0.8 a large effect. Where Cohen’s *d* was >1 the difference between the two means was considered larger than one standard deviation. Pearson correlations between all measures (ADOS, ADI-R, SCQ, MABC, BEERY VMI, SP, MoA, BPVS-III, Matrices) were calculated for the ASC, TD and combined ASC and TD groups. These correlations were then followed up by two separate supplementary analyses: stepwise linear regressions, using autism severity as outcome measures (ADOS-II, ADI-R and SCQ), and the MoA, SP, MABC, BPVS and Matrices scores as predictors; and an additional correlation of the separate components of autism, coordination and sensory measures. *Post hoc* power analyses on the multiple regression model were conducted using G * Power 3 (Faul et al., 2007) to compute the achieved statistical power for each model. Results showed all models, with the exception of Model 1 in the ADOS model (statistical power of 0.67), achieved statistical power >0.89 at the alpha level of *p* = 0.05 with a sample size of 18.

### Do Children with ASC Show Significant Sensory and Motor Difficulties?

Table 2 shows results of comparisons between the ASC and TD groups on all measures. Bonferroni corrected independent samples *t*-tests showed that children with ASC had significantly lower: receptive language ability (BPVS; *t*_(34)_ = −4.00, *p* < 0.001, *d* = 0.1.33); motor coordination skills (MABC; *t*_(34)_ = −4.56, *p* < 0.001, *d* = 1.52); sensory responsivity (SP; *t*_(34)_ = −7.69, *p* < 0.001, *d* = 2.56); (MoA; a sensorimotor component of the SP; *t*_(34)_ = 4.00, *p* < 0.001, *d* = 1.33); and higher parent reported autistic traits (SCQ scores; *t*_(24)_ = 7.33, *p* < 0.001, *d* = 2.44), than the TD group, all with medium to large effect sizes. After Bonferroni correction, there was a marginally non-significant group difference in visual-motor integration (BEERY VMI; *t*_(21)_ = 2.86, *p* = 0.009, *d* = 0.97).

**Table 2:** **Dependent variable descriptives and comparison of means**.

Group	BPVS standardized score	MABC composite total	Sensory profile total	SCQ score	BEERY VMI (ASC *N* = 17)	Modulation of Activity
ASC (*N* = 18)	88.56 ± 14.08	51.61 ± 15.69	228.22 ± 37.44	18.94 ± 7.94	84.24 ± 21.27	18.61 ± 3.17
Control (*N* = 18)	106.00 ± 12.02	74.28 ± 14.12	312.11 ± 27.19	3.83 ± 3.68	100.17 ± 8.93	23.11 ± 3.58
Difference	*t*_(34)_ = −4.00,	*t*_(34)_ = −4.56,	*t*_(34)_ = −7.69,	*t*_(24)_ = 7.33,	*t*_(21)_ = −2.86,	*t*_(34)_ = −4.00,
	*p*< 0.001,	*p*< 0.001,	*p*< 0.001,	*p*< 0.001,	*p* = 0.009,	*p*< 0.001,
	***d* = 1.33**	***d* = 1.52**	***d* = 2.56**	***d* = 2.44**	***d* = 0.97**	***d* = 1.33**

*Note: Effect size (Cohen’s d) is denoted in bold: 0.2 = small, 0.5 = medium, 0.8 = large. Bonferroni corrected p-value = 0.008*.

*Post hoc* independent bonferroni corrected *t*-tests then explored which specific components of the MABC and SP were significantly different between the TD and ASC groups. For the MABC, results showed that the manual dexterity composite was significantly different between groups with large effect (*t*_(28.96)_ = 5.562, *p* < 0.001, *d* = 1.85) and the balance composite was also significantly different between groups with large effect (*t*_(34)_ = 2.531, *p* = 0.016, *d* = 0.84). However the aiming and catching composite was not significantly different between groups (*t*_(34)_ = 1.999, *p* = 0.054, *d* = 0.67; Bonferroni corrected *p* = 0.017).

For the SP, results are shown in descending effect size according to Cohen’s *d*: poor registration (*t*_(25.92)_ = 7.273, *p* = <0.001, *d* = 2.42), emotional regulation (*t*_(34)_ = 6.788, *p* < 0.001, *d* = 2.26), low endurance (*t*_(24.42)_ = 5.858, *p* < 0.001, *d* = 1.95), attention composites (*t*_(25.91)_ = 4.738, *p* < 0.001, *d* = 1.58), sedentary (*t*_(34)_ = 3.709, *p* = 0.001, *d* = 1.24) and fine motor skill composites (*t*_(34)_ = 3.631, *p* = 0.001, *d* = 0.85) respectively were significantly different between groups with large effect. However, oral sensitivity (*t*_(34)_ = 2.809, *p* = 0.008, *d* = 0.94), sensory seeking (*t*_(27.39)_ = 2.392, *p* = 0.024, *d* = 0.80), and sensory sensitivity (*t*_(34)_ = 2.114, *p* = 0.042, *d* = 0.71) composites of the SP were not significantly different between groups after Bonferroni correction *p* = 0.006.

### Are Sensory Responsivity and Motor Coordination Associated with ASC Symptom Severity?

Pearson correlations were calculated in each group separately (ASC and TD) and with both groups combined (whole sample), between all variables (ADOS, ADI-R, SCQ, SP, MoA, MABC, BEERY VMI, BPVS and Matrices; Table 3).

**Table 3:** **Correlation analysis (*r*) for autism symptom measures, coordination and sensory responsivity in autism spectrum conditions (ASC), typically developing (TD) and combined ASC and TD group**.

	ADIR TOTAL	ADOS TOTAL	SCQ	MABC TOTAL	BEERY VMI	SP TOTAL	SP MoA	BPVS

	**ASC GROUP (*n* = 18)**							

ADOS TOTAL	0.566**
SCQ	0.831***	0.479*
MABC TOTAL	0.324	0.647**	0.241
BEERY VMI	0.112	0.336	0.015	0.611**
SP TOTAL	0.657**	0.256	0.836***	0.087	0.054
SP MoA	0.482*	0.518*	0.540*	0.477*	0.165	0.524*
BPVS	−737**	0.375	0.561**	0.464*	0.379	0.339	0.302
WASI MATRICES	0.076	0.031	0.036	0.495*	0.600**	0.065	0.246	0.297

	**TD GROUP (*n* = 18)**							

MABC TOTAL			0.264
BEERY VMI			0.351	0.043
SP TOTAL			0.419*	0.144	0.189
SP MoA			0.253	0.181	0.553**	0.461*
BPVS			0.024	0.299	0.493*	0.057	0.335
WASI MATRICES			0.366	0.353	0.407*	0.249	0.254	0.656**

	**Whole SAMPLE (*n* = 36)**							

MABC TOTAL			0.598***
BEERY VMI			0.379*	0.572**
SP TOTAL			0.893***	0.542***	0.404**
SP MoA			0.650***	0.442**	0.446**	0.692***
BPVS			0.645**	0.603**	0.542**	0.544**	0.534**
WASI MATRICES			0.328*	0.518**	0.568**	0.335*	0.376*	0.539**

Note: *p < 0.05, **p < 0.01, ***p < 0.001. Required r ≥ 0.522 for sample size and α 0.05 to achieve a Statistical Power = 0.8. Correlations in bold indicate results > required effect size.

Table 3 shows results of the correlation analysis. In the ASC group (*n* = 18), in addition to co-linearity between the three autism measures (ADI-R, ADOS and SCQ) and the two coordination measures (MABC and BEERY VMI), the SP and BPVS significantly correlated with the ADI-R (Current; SP *r* = −0.657, *p* = 0.002; BPVS *r* = −0.737, *p* < 0.001) and SCQ (SP *r* = −0.836, *p* < 0.001; BPVS *r* = −0.561, *p* = 0.008) with medium to large effect. The MABC significantly correlated with the ADOS-2 (*r* = −0.647, *p* = 0.002) with medium to large effect, and the Matrices and BPVS with a small to medium effect (Matrices *r* = −0.495, *p* = 0.018; BPVS *r* = −0.464, *p* = 0.026). The Matrices also demonstrated correlation with the BEERY VMI in this group (*r* = −0.600, *p* = 0.005) with medium to large effect. In the TD group (*n* = 18) the SP showed some correlation with the SCQ with small to medium effect) *r* = −0.419, *p* = 0.042). The Matrices and BPVS (*r* = −0.656, *p* = 0.002) significantly correlated with medium to large effect. The BEERY VMI also demonstrated some correlation with the BPVS and Matrices with small to medium effect (Matrices *r* = −0.407, *p* = 0.047; BPVS *r* = −0.493, *p* = 0.019). When the TD group were added to the ASC group (*n* = 36) both the SP and MABC significantly correlated with autism severity levels in the SCQ (SP *r* = −0.893, *p* < 0.001; MABC *r* = −0.598, *p* < 0.001). Furthermore, when the groups were combined the SP also correlated with the MABC and BEERY VMI (MABC *r* = −0.542, *p* < 0.001; BEERY VMI *r* = −0.404, *p* = 0.008). The BPVS showed medium effect correlations across all variables (SCQ *r* = −0.645, *p* < 0.001; MABC *r* = −0.603, *p* < 0.001; BEERY VMI *r* = −0.542, *p* < 0.001; SP *r* = −0.544, *p* < 0.001; MoA *r* = −0.534, *p* < 0.001) and the Matrices demonstrated small to medium effect correlations across all variables when both groups were combined (SCQ *r* = −0.328, *p* = 0.025; MABC *r* = −0.518, *p* = 0.001; BEERY VMI *r* = −0.568, *p* < 0.001; SP *r* = −0.335, *p* = 0.023; MoA *r* = −0.376, *p* = 0.012; BPVS *r* = −0.539, *p* < 0.001).

The MoA in the ASC group (MoA; an independent sensorimotor variable within the SP) significantly correlated with all three ASC measures (ADI-R *r* = 0.482, *p* = 0.021; ADOS *r* = 0.518, *p* = 0.014; SCQ *r* = 0.540, *p* = 0.010) and the MABC (*r* = 0.477, *p* = 0.023). In the TD the MoA significantly correlated only with the BEERY VMI (*r* = 0.553, *p* = 0.009). However, MoA demonstrated significant correlation with the SCQ (*r* = −0.650, *p* < 0.001) and BPVS (*r* = 0.534, *p* < 0.001) with medium to large effect when the TD group’s data were added to the ASC group’s data, and small to medium effect with the MABC Total (*r* = 0.442, *p* = 0.004) BEERY VMI (*r* = 0.446, *p* = 0.004) and Matrices (*r* = 0.376, *p* = 0.012).

In the ASC group, supplementary analyses were then performed using stepwise multiple regression with autism severity as outcome measures (ADOS-II, ADI-R Current and SCQ), and the SP, MABC, BPVS and Matrices scores as predictors. BEERY VMI was not included as a predictor in this analysis, due co-linearity with the MABC. Therefore if included, the BEERY VMI would have reduced the statistical power of this analysis to find a significant effect of motor coordination with autism symptom severity.

**Figure 1:**
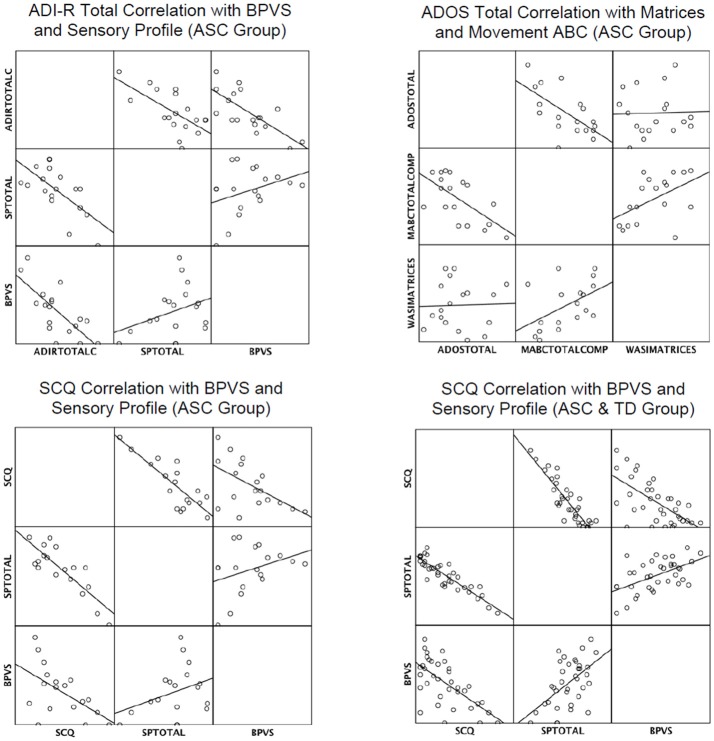
**Matrix scatterplots to show correlations in autism measures and retained predictors in multiple regression analyses.** *Key: ADIRTOTALC, ADI-R TOTAL Current; SPTOTAL, Sensory Profile Total; MABCTOTALCOMP, MovementABC Total Composite Score*.

Table 4 shows results of the supplementary stepwise regressions. In the ASC group the BPVS and SP were retained as significant predictors of ADI-R Total (Current); the MABC and Matrices were retained as a significant predictors of ADOS-II total; and the SP and BPVS were retained as significant predictors of the SCQ. The SP and BPVS were also significant predictors of autism levels in the SCQ when the TD group was added to the ASC group. In summary, results showed that the SP scores significantly predicted parent reported autism symptom severity (ADI-R and SCQ), and the MABC measures significantly predicted an in-person measure of autism severity (ADOS-II). The BPVS and Matrices predicted some, but not all, of the parent reported social and communication skills in ASC. Figure 1 demonstrates visually how the predictors correlated with the autism measures.

**Table 4:** **Stepwise multiple regressions for autism symptom measures in ASC group**.

Step	Variable	*B*	*SE B*	*B*	Cum *R*^2^

**ADI-R Total (Current) (*n* = 18)**					

1	Constant	70.31	10.16
	BPVS	0.50	0.11	0.74***	0.74
2	Constant	87.54	9.67
	BPVS	0.39	0.10	0.58**	0.85
	SP TOTAL	0.12	0.04	0.46**

**ADOS-II Total (*n* = 18)**					

1^∧^	Constant	24.00	3.39
	MABC TOTAL	0.21	0.06	0.65**	0.65
2	Constant	11.44	5.97
	MABC TOTAL	0.29	0.06	0.87***	0.77
	Matrices	0.18	0.08	0.47*

**SCQ (*n* = 18)**					

1	Constant	59.40	6.72
	SP TOTAL	0.18	0.03	0.84***	0.70
2	Constant	69.93	7.23
	SP TOTAL	0.16	0.03	0.73***	0.79
	BPVS	0.18	0.07	0.31*

**SCQ with ASC and TD group (*n* = 36)**					

1	Constant	55.67	3.89
	SP TOTAL	0.16	0.01	0.89***	0.80
2	Constant	63.32	4.59
	SP TOTAL	0.14	0.02	0.77***	0.83
	BPVS	0.14	0.05	0.23*

Note: *p < 0.05, **p < 0.01, ***p < 0.001. All Models with the exception of 1^∧^ achieved >0.89 Statistical Power for a sample size of 18 and α 0.05 1^∧^ Statistical Power = 0.67.

Table 5 shows results of a separate linear regression analysis for the main effect of MoA (an independent sensorimotor variable within the SP), predicting ASC symptom severity (ADOS, ADI-R and SCQ). Results demonstrated that MoA significantly predicted ASC symptom severity both in the ASC group across all diagnostic measures (ADOS, ADI-R and SCQ), and when the TD group’s SCQ data were added to the ASC group’s SCQ data.

**Table 5:** **Overall model fit, beta values, *t*-values, and *p*-values are displayed for each diagnostic measure**.

ASC symptom measure	Modulation of activity			
ADIR-TOTAL	*R*^2^ = 0.321	*F*_(1,16)_ = 7.550	β = −0.566, *t* = −2.748	*p* = 0.014
ADOS-TOTAL	*R*^2^ = 0.264	*F*_(1,16)_ = 5.751	β = −0.514, *t* = −2.398	*p* = 0.029
SCQ	*R*^2^ = 0.291	*F*_(1,16)_ = 6.569	β = −0.540, *t* = −2.563	*p* = 0.021
SCQ with ASC and TD group (*n* = 36)	*R*^2^ = 0.422	*F*_(1,34)_ = 24.849	β = −0.650, *t* = −4.985	*p* = 0.0001

All values indicate that Modulation of Activity is also a significant predictor of autism severity for each diagnostic measure. Note: Using G* Power 3 (Faul et al., 2007) an effect size (β) of 0.522 was considered significant at 0.8 Power and 18 ppts).

Table 6 shows results of a further analysis in the ASC group, where individual ASC measures, coordination and sensory components were correlated in order to identify any specific links. Measures were divided into motor, sensory and sensorimotor. Results demonstrate significant correlations with autism measures in the sensorimotor components alone. Furthermore, vestibular (balance), auditory, tactile and oral motor responsivity appear to be correlated with more than one autism component.

**Table 6:** **Correlation analysis (*r*) for specific autism components, coordination components and sensory components in ASC group (*n* = 18)**.

Measure	ADIRC	ADIRRSI	ADIRRSB	ADOSC	ADOSRSI	ADOSRRB	SCQ

	**Motor Measures**						

BEERY MC	0.207	0.009	0.032	0.252	0.380	0.032	0.032

	**Sensory Measures**						

BEERY VP	0.195	0.109	0.074	0.338	0.282	0.145	0.109
SPVIS	0.280	0.338	0.390	0.280	0.032	0.134	0.338

	**Sensorimotor Measures**						

BEERY VMI	0.093	0.106	0.301	0.422*	0.372	0.057	0.015
MABCMD	0.152	0.331	0.006	0.595**	0.535*	0.200	0.256
MABCAC	0.253	0.076	0.174	0.309	0.117	0.188	0.092
MABCB	0.322	0.448*	0.322	0.531*	0.523*	0.344	0.238
SPAUD	0.652**	0.694**	0.521*	0.307	0.178	0.251	0.861***
SPVEST	0.201	0.179	0.590**	0.173	0.053	0.084	0.381
SPTOUCH	0.334	0.522*	0.626**	0.506*	0.365	0.069	0.615**
SPORAL	0.576**	0.310	0.693**	0.217	0.197	0.464*	0.574**

Note: *p < 0.05, **p < 0.01, ***p < 0.001. Required r = >0.522 for sample size and α 0.05 to achieve a Statistical Power = 0.8. Correlations in bold indicate results > required effect size. Key: ADIRC, ADI-R Communication; ADIRRSI, ADI-R Reciprocal Social Interaction; ADIRRSB, ADI-R Restricted Stereotyped Behaviors. ADOSC, ADOS Communication; ADOSRSI, ADOS Reciprocal Social Interaction; ADOSRB, ADOS Stereotyped Repetitive Behaviors. BEERY MC, BEERY VI Motor Coordination; BEERY VP, BEERY VI Visual Perception; BEERY VMI, BEERY VI Visual Motor Integration. MABCMD, MABC Manual Dexterity; MABCAC, MABC Aiming and Catching; MABCB, MABC Balance. SPVIS, Sensory Profile Visual; SPAUD, Sensory Profile Auditory; SPVEST, Sensory Profile Vestibular; SPTOUCH, Sensory Profile Tactile; SPORAL, Sensory Profile Oral Motor.

## Discussion

The current study investigated whether children with ASC show significant difficulties in sensory responsivity and motor coordination compared to children without ASC, and whether these difficulties were significantly associated with ASC symptom severity. Results showed the children with ASC had significant motor coordination, sensory responsivity and receptive language difficulties compared to age and non-verbal IQ matched children without ASC. Analysis of the different subcomponents of sensory responsivity and motor coordination difficulties showed that the children with ASC had specific impairments in particular domains; the more significant sensory responsivity impairments in the ASC group were in poor registration, low endurance, emotional regulation, attention, sedentation and fine motor skills. The more significant motor coordination impairments were in manual dexterity and balance. These results confirm previous reports showing significant difficulties in sensory responsivity and motor coordination in those with ASC in comparison to TD controls, such as Matson et al. (2010) and MacNeil and Mostofsky (2012), respectively. Our analysis of the key areas of difficulty within these respective domains also mirror previous research, such as that by Tomchek and Dunn (2007), where 90% of ASC participants demonstrated a significant difference when compared to TD controls in the underesponsive domains of the SP and Siaperas et al. (2012) who showed that individuals with ASC presented with difficulties in both balance and fine motor skills.

Results also showed significant correlations between sensory responsivity, motor coordination and the severity of ASC symptoms in the ASC, TD and combined ASC and TD groups. Results demonstrated possible co-linearity between the BPVS and communication measures of autism (ADI-R and SCQ) and the Matrices and BEERY VMI, which are both measures of spatial reasoning. However, the correlation between the BPVS and Matrices was only significant, with medium to large effect, in the TD group: not the ASC group, perhaps indicating an association between non-verbal and verbal performance in this group alone. We were interested in whether sensory and motor difficulties could account for ASC symptom severity in ASC, TD and combined groups and therefore followed up these significant correlations with supplementary regression analyses, controlling for group differences in receptive language and non-verbal IQ. Results showed that difficulties in sensory responsivity and motor coordination in both the ASC and combined ASC and TD groups significantly predicted severity of ASC symptoms, over and above receptive language ability and non-verbal IQ. However, the presence of a significant predictive relationship depended on the type of measures used. Specifically, sensory responsivity difficulties significantly predicted the level of ASC symptoms in both parental report measures of ASC (ADI-R and SCQ), whilst motor coordination difficulties significantly predicted the level of autism symptoms measured by the ADOS-II, an in person measure of ASC through examiner observation. One could argue that this correlation between the parental measures and the observational measures is unsurprising. However, these measures were not analogous. For example, the ADOS-2 does not specifically measure coordination and the ADI-R does not specifically measure sensory responsivity. Therefore these correlations were unlikely to have occurred if sensory responsivity or motor coordination skills were in no way related to severity of autism symptoms. Additionally, a medium to large effect correlation between the SCQ, SP and MABC was apparent when ASC and TD data were merged. MoA, a subset of the SP that measures activeness and exploration, giving an indication of sensorimotor integration (sensory environment governing movement), was also associated with ASC severity across all diagnostic measures (ADOS-II, ADI-R and SCQ).

The fact that the presence of a predictive relationship between sensory responsivity and motor coordination and autism symptoms differed according to the diagnostic measure used (ADOS or ADI-R), could have also been due to the differing properties of these instruments. For example, the ADOS-2 is a short time window of current behavior, approximately 1 h interaction with an examiner. This gives few opportunities for the child to display sensory abnormalities (except with a spinning disk and pin art during the break), or poor attention given the 1:1 nature of the interaction. In addition scoring opportunities of sensory responsivity is limited; specifically the ADOS-2 only has two items, which take into account sensory abnormalities such as seeking behaviors. Hence, it is unlikely that the type of behaviors seen in the ADOS-2 would correlate with the SP, which takes into account sensory responsivity across a variety of context and time points in children with ASC, particularly as this measure was significantly characterized by poor registration and attention. In contrast, the ADI-R is a parent-report measure, with current behavior ranging from yesterday to months ago, with more emphasis and opportunity for parents to report on sensory and attention difficulties compared to the ADOS-2. Furthermore, the ADI-R places less emphasis on non-verbal language and gesture based on the proportion of algorithm questions (the ADOS-II having 50% of codes based on social interaction and the ADI-R Current algorithm 38%). Given that the ADOS-2 and ADI-R give different priorities to different behaviors and thus are utilized together as diagnostic instruments, could explain why sensory and motor skills are associated with different autism measures.

The results from the *post hoc* detailed analysis of the relationships between motor, sensory and sensorimotor abilities and the specific components of ASC, demonstrated a significant sensorimotor association with ASC severity across the ASC domains. Moreover, the specific sensorimotor domains correlated are all instrumental to somatosensory perception. For example tactile, acoustic and vestibular information respond using mechanoreceptors to skin or head displacement (Yeomans et al., 2002) and somatosensory awareness also occurs orally to ensure alertness to objects in the mouth (Haggard and de Boer, 2014).

The strong correlation between both sensory responsivity and motor coordination with the SCQ; results from the supplementary analysis showing that sensorimotor skills, MoA, sensory responsivity and motor coordination all predicted measures of ASC symptoms over and above receptive language and non-verbal IQ both in the ASC group alone, and the combined ASC and TD groups; and the specific correlation of the senses involved in somatosensory perception such as touch, vibration and pressure, with the different components of autism, would suggest that sensory and motor difficulties may together, in the form of sensorimotor integration, significantly impact the severity of ASC symptoms. These results are consistent with previous research, such as that by Gowen and Hamilton (2013), who suggested that atypical sensory input and variability in motor deployment simultaneously could potentially impact on severity of ASC symptoms such as social and communication skills. Additionally, Ozonoff et al. (2008) demonstrated atypical MoA and exploration in children with ASC from an early age by showing toddlers with ASC who spent significantly more time spinning and rotating objects when compared to their TD peers, and Kawa and Pisula (2010) showed 4–5 year olds differed in both object exploration and time spent in more visually complex zones, which was significantly decreased in comparison to other areas. Research has also shown that this reduced exploration and activity shown in ASC individuals is linked to the cerebellum, an area of the brain known to be linked to sensorimotor integration (Glickstein, 1998), and that measures of decreased activity significantly correlates with cerebellar hypoplasia (decreased cerebellum size) in ASC (Pierce and Courchesne, 2001).

Recent research has also shown that these difficulties with sensorimotor integration are particularly key in ASC. For example, Whyatt and Craig (2012) found that the main motor skill deficits in ASC are those that demand more perception-action coupling and anticipatory control such as with manual dexterity. This finding is consistent with the current study’s results, which showed a specific difficulty in manual dexterity in the ASC compared to the TD group. Participants with ASC also had significantly more difficulty with the MABC, compared to the BEERY VMI. As most of the tasks in the MABC require ability to time movements in response to sensory feedback such as when catching a ball or timed peg turning, as opposed to VMI alone as with the BEERY VMI, this suggests more specific issues with temporal sensorimotor adjustment as opposed to VMI in ASC (Price et al., 2012; Bo et al., 2014). Again, these anticipatory and predictive adjustments of motor programs that are paramount to movement occur in the cerebellum (Koziol et al., 2012), an area of the brain where abnormalities are consistently found in individuals with ASC (Fatemi et al., 2012).

Results from the current study suggest that difficulties in sensorimotor skills are significantly associated with the social communication difficulties and behaviors characteristic of ASC. It would be beneficial to identify which aspect of the sensorimotor motor chain may be affected in ASC. Vandenbroucke et al. (2009) suggested that with significant practice, individuals with ASC could create successful feedforward motor programmes. Larson et al. (2008) reinforced this finding by showing that the mechanisms for acquisition and adaptation of feedforward programs in children with ASC were equal to TD children. Studies have also highlighted difficulties in incorporating sensory input, such as environmental cues, into motor planning (Gowen and Miall, 2005; Gowen et al., 2008; Dowd et al., 2012). Nazarali et al. (2009) also found that individuals with ASC had difficulty reprogramming pre-planned movement. This evidence suggests that a feedforward program can be established by individuals with ASC, but once established, the essential environmental information that is usually used to fine-tune any movement is not utilized effectively. Such physical difficulties in adapting to environmental prompts from an early age would likely have huge impact on an individual’s ability to detect, understand and react to social information appropriately. Furthermore, exploration would likely be decreased which in turn would instigate a negative cycle: limited exploration, limited sensory feedback, limited sensorimotor planning, cerebellar hypoplasia, decreased exploration and so on.

A limitation of the current study is that it includes a reasonably small sample (18) in each group. However, the analysis demonstrated medium to large effect sizes alongside Bonferroni correction, and thus demonstrated significant effects. A power analysis also showed that 18 participants had sufficient statistical power to detect a medium to large effect in our supplementary regression analysis. Although we would be unable to detect smaller effect sizes, results from our correlational and supplementary regression analyses nevertheless showed significant associations between sensory, motor and sensorimotor skills, and severity of ASC symptoms, over and above receptive language and non-verbal IQ, with medium-large effect sizes. We also replicated these effects found in the correlation and supplementary regression analyses in a larger combined ASC and TD sample of 36 individuals. This suggests that difficulties in these areas are significantly associated with severity of ASC symptoms. A questionnaire-based study exploring sensorimotor questions would be advantageous in order to help verify our current findings in a larger sample. Another limitation of this study is that we cannot infer causality using our correlational design. Longitudinal studies are needed to explore the causal relationship between early sensory and motor difficulties and their impact on later social and communicative functioning. Intervention studies that demonstrate that improvement of sensorimotor abilities also improve social and communicative abilities in ASC individuals are also important in order to establish causation. Additionally, the SP does not solely look at sensory perception, items also capture behavioral outcomes and emotional responses, such as “has temper tantrums”. Many of these are part of, or co-morbid with ASC. Finally, the difference in matrices performance between the groups approached significance (without Bonferroni correction), though it was clearly smaller in effect size than the differences found in the MABC and SP. Our supplementary regression analysis also controlled for group differences in receptive language and non-verbal IQ, and still found significant results showing associations between sensory, motor, and sensorimotor skills and ASC symptoms. Hence, differences in verbal and non-verbal performance between groups were unlikely to have invalidated the results.

In conclusion, this study suggests that the social communication, interaction and behavioral difficulties that children with ASC experience can be predicted by difficulties in sensorimotor abilities, suggesting possible impairment of sensorimotor integration. Current diagnostic criteria for ASC (DSM-5, American Psychiatric Association, 2013) incorporate difficulties in sensory responsivity and motor movements, although it is not clear whether these are symptomatic of, or instrumental to autism. This study has not included causation, but perhaps it will prompt future research into sensorimotor causality by exploring: the nature of the sensorimotor difficulties in ASC compared to other related conditions (e.g., ADHD and Dyspraxia) to ascertain how sensorimotor integration differs across syndromes; micro-movement methodology (Torres et al., 2013) to further investigate disruptions in sensorimotor integration; and whether integrated sensory activities and temporal spatial coordination activities increase reciprocal social interaction capability. Furthermore, if a child’s 3-year developmental review presents weaknesses in sensorimotor skills, alongside intervention this information should be noted and employed as an important assessment and possible ongoing observation tool for ASC diagnosis purposes and measures of severity.

## Author Contributions

PH: developed study topic and designed the study, literature search, recruited participants, collated and analyzed data, wrote the manuscript and completed the tables. SC: supported design of the study, collated and analyzed data, gave critical feedback, wrote the manuscript. TT: analyzed data, gave critical feedback, wrote the manuscript. FM: collated data, critical revision of the manuscript. All authors read and approved the final manuscript.

## Conflict of Interest Statement

The authors declare that the research was conducted in the absence of any commercial or financial relationships that could be construed as a potential conflict of interest.
